# Systematic Analysis of bHLH Transcription Factors in Cassava Uncovers Their Roles in Postharvest Physiological Deterioration and Cyanogenic Glycosides Biosynthesis

**DOI:** 10.3389/fpls.2022.901128

**Published:** 2022-06-16

**Authors:** Feifei An, Xinhui Xiao, Ting Chen, Jingjing Xue, Xiuqin Luo, Wenjun Ou, Kaimian Li, Jie Cai, Songbi Chen

**Affiliations:** ^1^Tropical Crops Genetic Resources Institute, Chinese Academy of Tropical Agricultural Sciences/Key Laboratory of Ministry of Agriculture for Germplasm Resources Conservation and Utilization of Cassava, Haikou, China; ^2^School of Life Sciences, Hainan University, Haikou, China; ^3^Postgraduate Department, Hainan Normal University, Haikou, China

**Keywords:** cassava, *bHLH* gene family, transcription factors, postharvest physiological deterioration, cyanogenic glucosides

## Abstract

The basic helix-loop-helix (bHLH) proteins are a large superfamily of transcription factors, and play a central role in a wide range of metabolic, physiological, and developmental processes in higher organisms. However, systematic investigation of *bHLH* gene family in cassava (*Manihot esculenta* Crantz) has not been reported. In the present study, we performed a genome-wide survey and identified 148 *MebHLH*s genes were unevenly harbored in 18 chromosomes. Through phylogenetic analyses along with *Arabidopsis* counterparts, these *MebHLHs* genes were divided into 19 groups, and each gene contains a similar structure and conserved motifs. Moreover, many *cis*-acting regulatory elements related to various defense and stress responses showed in *MebHLH* genes. Interestingly, transcriptome data analyses unveiled 117 *MebHLH* genes during postharvest physiological deterioration (PPD) process of cassava tuberous roots, while 65 *MebHLH* genes showed significantly change. Meanwhile, the relative quantitative analysis of 15 *MebHLH* genes demonstrated that they were sensitive to PPD, suggesting they may involve in PPD process regulation. Cyanogenic glucosides (CGs) biosynthesis during PPD process was increased, silencing of *MebHLH72* and *MebHLH114* showed that linamarin content was significantly decreased in the leaves. To summarize, the genome-wide identification and expression profiling of *MebHLH* candidates pave a new avenue for uderstanding their function in PPD and CGs biosynthesis, which will accelerate the improvement of PPD tolerance and decrease CGs content in cassava tuberous roots.

## Introduction

The basic helix-loop-helix (bHLH) proteins are a large superfamily of transcription factors, and widely distributed in plants, fungi and animals (Steidl et al., 2000; Carretero-Paulet et al., 2010). The bHLH domain contains a basic region and a HLH (helix-loop-helix) region, and the N-terminal basic region directly followed by the HLH domain (Sharker et al., 2020). More than 50% of bHLHs in plant possess a conserved HER motif in order to binding and regulating their target genes (Toledo-Ortiz et al., 2003). The basic region, is a DNA-binding region that allows HLH proteins to bind to E-box (CANNTG) (Massari and Murre, 2000). Basic helix-loop-helix transcription factors play crucial roles in plant growth and development including morphogenesis (Gangappa and Kumar, 2017; Ballester et al., 2021), iron homeostasis (Li et al., 2019), flower and fruit development (Sun K. et al., 2019), stomatal initiation (Raissig et al., 2016), root vascular cell proliferation (Ohashi-Ito and Fukuda, 2016), grain yield (Luo et al., 2013), secondary metabolites biosynthesis, such as anthocyanin (Zhao R. et al., 2020; Li et al., 2021), also in the stress tolerance, for instance, drought (Verma et al., 2020; Zhao Q. et al., 2020), salt (Liu et al., 2021; Yu C. et al., 2021), cold (Luo et al., 2020; Shen et al., 2021), heavy metal toxicity (Sun W. et al., 2019), and osmotic stress (Yang et al., 2016). Despite some bHLHs functions have been elaborated, most plant bHLHs functions are still unclear, especially in tuber crops.

Cassava (*Manihot esculenta* Crantz) is a staple food in the tropics and sub-tropics regions, which has high starch content and strong adaptability (An et al., 2019a). However, cassava roots storage is limited by postharvest physiological deterioration (PPD), it begins in 24 hours and then the roots became unpalatable and unmarketable rapidly (Zainuddin et al., 2017). PPD is a wound response of cassava roots during the harvest time, which is not due to microbial infection (Udogu et al., 2021). Accompanied with PPD occurs, an oxidative burst is initiated, and then following with the accumulation of secondary metabolites (Reilly et al., 2004). The process of PPD involved in reactive oxygen species (ROS), calcium signal transduction, cyanogenic glucosides biosynthesis, starch degradation, phenylpropanoid biosynthesis, N-glycosylation modification and programmed cell death (PCD) (Owiti et al., 2011; Djabou et al., 2017; Qin et al., 2017; An et al., 2019b). Many bHLHs are involved in abiotic stress processes in plants, however, its role in PPD process is still unknown. In the present study, the *bHLH* genes of cassava (*MebHLH*) was systematically identified and characterized, also compared with the *bHLHs* homology of *Arabidopsis thaliana*. Sequence comparison revealed that the presence and distribution of duplicated genes occurred among *MebHLH* genes. To identify *MebHLH* candidate genes associated with PPD resistance, the expression patterns of *MebHLH* genes were analyzed using transcriptome data of different PPD materials, and then the gene expression profiling of selected *MebHLH* candidate genes were verified by qRT-PCR. In addition, the cyanogenic glycosides content was detected during PPD process, and the function of *MebHLHs* in cyanogenic glycosides biosynthesis was confirmed. This study provides comprehensive information about cassava *bHLH* genes family, as well as a basis for further analysis of novel *MebHLH* candidate genes, which may be useful for improving PPD resistance and cyanogenic glycosides biosynthesis in cassava.

## Materials and Methods

### Identification of *MebHLH* Genes in Cassava Genome

The whole genome sequences (v. 8.0) of cassava were downloaded from the phytozome database^1^. And the Hidden Markov Model (HMM) profile of bHLH (PF0001) was retrieved from Pfam (Finn et al., 2016)^2^. The HMMER program (Johnson et al., 2010) was used to search for bHLH protein in cassava genome, then all the putative proteins were further confirmed by the Pfam and SMART database (Letunic et al., 2021)^3^. The MW (molecular weight) and p*I* (theoretical isoelectric point) of these identified bHLH protein were predicted by ExPASy (Artimo et al., 2012)^4^. Finally, according to their locations on the chromosomes, we named the sequences with complete bHLH domains in order.

### Chromosomal Mapping, Gene Structure and Conserved Motif Analysis

The gene structures were performed by the CDS program and DNA sequences of *MebHLH* genes were visualized using the GSDS2.0 (Hu et al., 2015)^5^. The locations of these *MebHLH* genes were determined by querying the cassava genome. Additionally, chromosomal mapping was constructed by MapChart program (v. 2.32) (Voorrips, 2002). In addition, the online software MEME (Bailey et al., 2015)^6^ was used to identify the conserved motifs among all *MebHLH* genes, all the parameters were default setting except the number of motifs was set to 10.

### *Cis*-Acting Regulatory Element Analysis

The 1,500 bp upstream sequences of transcription start site ATG were extracted as the promoter sequence and screened them using PlantCare (Lescot et al., 2002)^7^ and PLACE (Higo et al., 1999)^8^ databases to identify cis-acting regulatory elements.

### Phylogenetic Analysis, Gene Duplication, Multiple Alignments and Synteny Analysis

MEGA X (Kumar et al., 2018)^9^ was used to multiple sequence alignment analysis of bHLH domain sequences. A phylogenetic tree was constructed with the neighbor-joining method and the parameters were poisson correction, pairwise deletion, and 1,000 bootstrap replicates. Gene duplication analysis mainly adopted two criteria, that is, the aligned sequence length was more than 75% of the long gene, and the aligned region similarity was greater than 75% (Vatansever et al., 2016). Clustal X (V.2.0) program (Thompson et al., 1997) was used to compare the coding sequences of repeated genes, and KaKs was used to calculate the non-synonymous replacement rate (Ka) and synonymous replacement rate (Ks). Calculator package (Zhang et al., 2006) via model averaging. The approximate date of duplication events (million years ago, Mya) was estimated by the formula T D Ks = 2λ × 10^–6^, on the basis of molecular clock rate of 2.6 × 10^–9^ substitutions/synonymous site for cassava. Circos program (Krzywinski et al., 2009) was used to illustrate the relationships of duplicated genes. The synteny of *bHLH* genes between cassava and *A. thaliana* was detected by MCScanX (Wang et al., 2012).

### RNA-Seq Data Analysis During Postharvest Physiological Deterioration

The tuberous roots of South China 9 (SC9) were harvested after planting for 10 months, and then cultivated in a chamber at 26 °C and a 16/8 h photoperiod (day/night). After 3 days, the tuberous roots with different PPD degree were selected as materials for transcriptome analysis. The degree of PPD was classified into 4 levels, in which No PPD was marked as I and PPD score was 0, extremely slight PPD was marked as II and PPD score was 3.2%, slight PPD was marked as III and PPD score was 10.6%, severe PPD was marked as IV and PPD score was 32.5% (Figure 6A), PPD score was according to Pahmawati et al., 2022. All samples were collected and frozen in liquid nitrogen immediately and stored at −80°C for further analysis. The platform for transcriptome sequencing was Illumina (HiSeq X-Ten). Three biological repeats were implemented. The heat maps were drawn using the heatmap software Mev.

**FIGURE 1:**
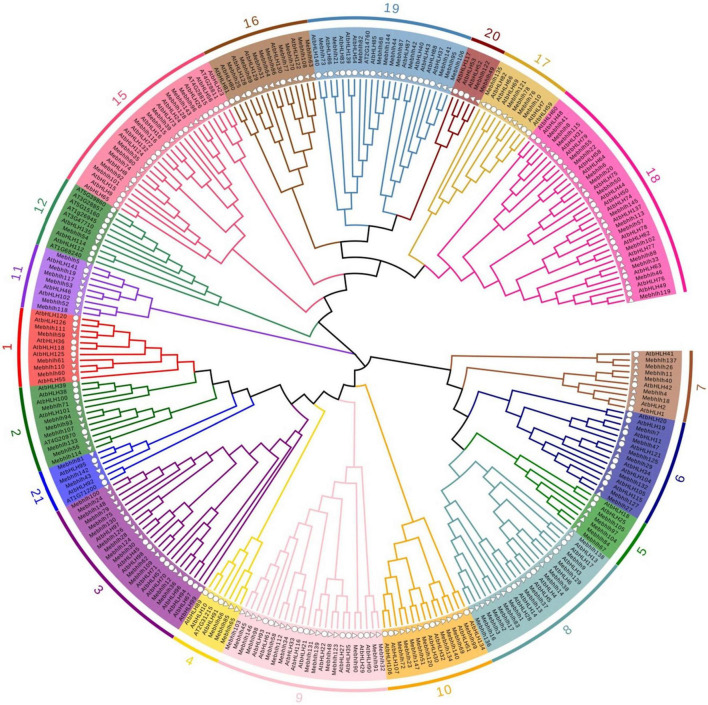
Phylogenetic tree of the 148 MebHLH proteins. Multiple sequence alignment of bHLH domain sequences of *M. esculenta* and *A. thaliana* was performed using Clustal W. MEGA X was used to construct the neighbor-joining (NJ) tree with 1,000 bootstrap replicates. Δ indicate bHLHs in *M. esculenta*, indicate bHLHs in *A. thaliana.*

**FIGURE 2:**
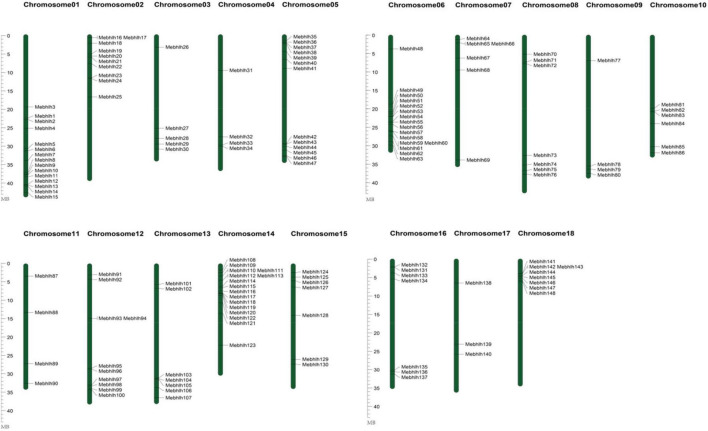
Chromosomal locations of the *MebHLH* genes. The color gradient provided a scale from 0 to 100% for assessing the magnitude of the parameters. Blue bars denote the *Manihot esculenta* chromosome. Scale bar on the left indicates the chromosome lengths (Mb).

**FIGURE 3:**
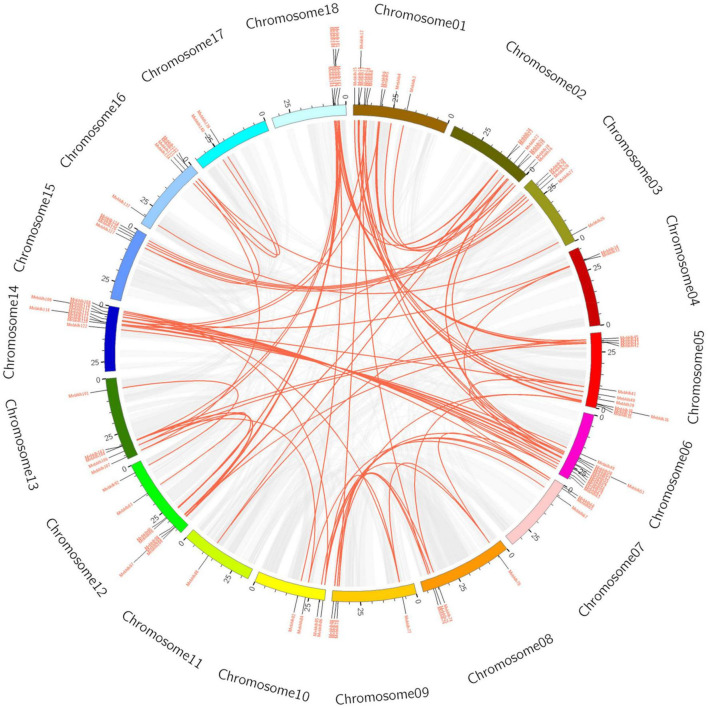
Circos diagram of the *MebHLH* duplication pairs in *M. esculenta*. *MebHLH* duplication pairs are linked with red lines. Scale bar marked on the chromosome indicating chromosome lengths (Mb).

**FIGURE 4:**
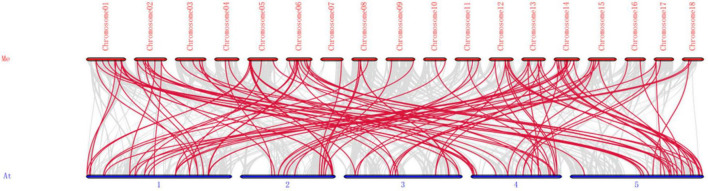
Synteny analysis of *bHLH* genes between *M. esculenta* and *A. thaliana.* Me indicates *M. esculenta*, At indicates *A. thaliana*. Gray lines in the background represent the collinear blocks within the genomes of *M. esculenta* and *A. thaliana*, while the red lines show the collinear *bHLH* gene pairs.

**FIGURE 5:**
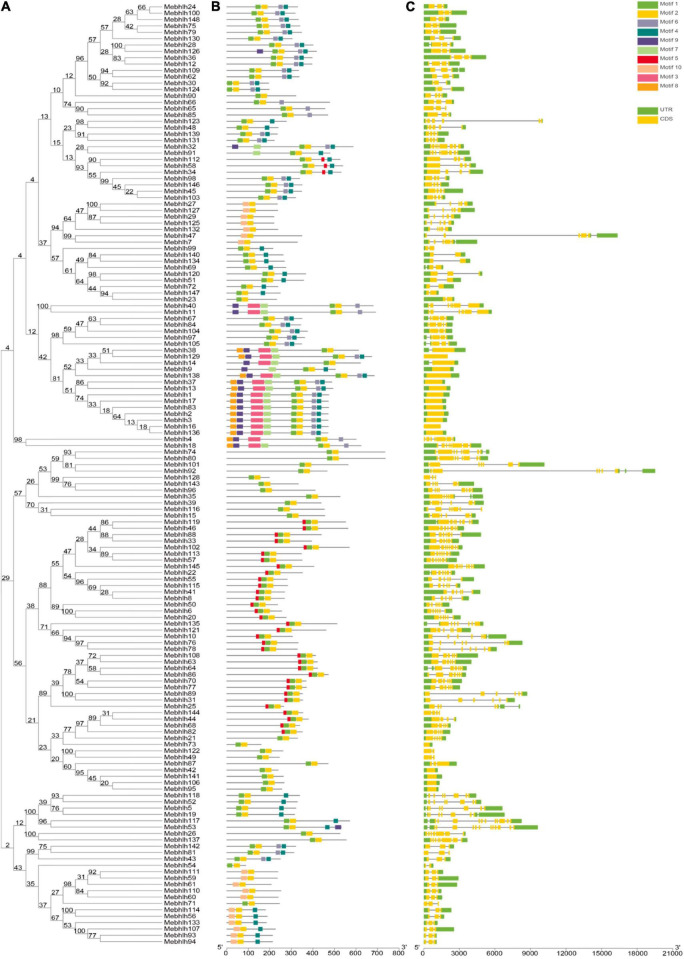
Putative conserved motifs and gene structures of the *MebHLH* genes. **(A)** Multiple sequence alignment of bHLH domain sequences of *M. esculenta* was performed using ClustalW. The neighbor-joining (NJ) tree was constructed using MEGA X with 1,000 bootstrap replicates. **(B)** Conserved motif. MEME analysis revealed the conserved motifs of the MebHLH proteins. The colored boxes on the right denote 10 motifs. **(C)** Gene structure. The yellow boxes, black lines, and green boxes represent exon, intron, and UTR (untranslated region), respectively.

**FIGURE 6:**
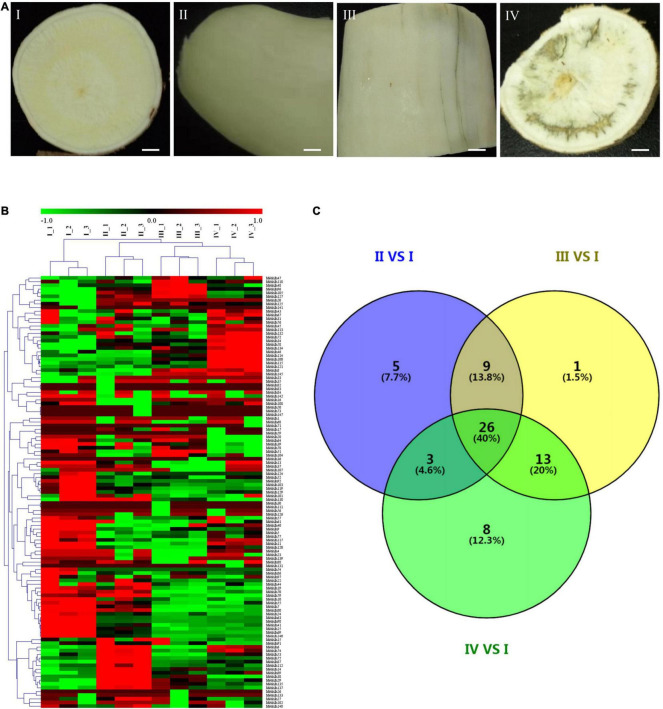
Expression profiles of *MebHLH* genes in tuberous roots during PPD process. **(A)** Different PPD degree samples collected from SC9 tuberous roots was classified into 4 levels. I, PPD score was 0, II, PPD score was 3.2%, III, PPD score was 10.6%, IV, PPD score was 32.5%. Bars = 5 mm. **(B)** The heat map was generated by Mev software. The expression data were gene-wise normalized and hierarchically clustered with average linkage. The bar on the right of the heat map indicates relative expression values. Values 1, 0, -1 represent high, intermediated, and low expression, respectively. **(C)** Venn diagram of differential expression *MebHLHs* in three comparison pairs. The sample with 0 PPD score was used as control.

### Determination of Cyanogenic Glucosides by HPLC-ELSD

Cyanogenic Glucosides (CGs) including linamarin and lotaustralin in cassava tuberous roots were measured as described in Wang et al., 2022, while CGs in cassava leaves were detected with minor modification. The HPLC-ELSD conditions are as follows: Waters Atlantic C18 column (4.6 mm x 150 mm, 5 μm), The column temperature is 25°C, the binary gradient elution is adopted, and the mobile phase A is 80% acetonitrile/water (V/V) containing 0.1% formic acid (V/V), B is 0.1% (V/V) aqueous formic acid solution. At the beginning, A is 10%, B is 90%, and at 15 min, both A and B are 50%; at 18 min, A is 100%, and maintained for 2 min; at 22 min, A changes 10%, and B% is 90%; balance the column for 5 min, and inject the next sample. The flow rate is 0.6 mL/min; drift tube temperature is 95°C, atomization temperature is 80°C, air flow rate is 2.0 L/min; gain is 3; injection volume is 10 μL. The contents of two CGs in the sample were calculated by external standard method. Linamarin and lotaustralin content (mg/kg) = C * V/1000M. C-the concentration of Linamarin or lotaustralin found in the standard curve, (mg/L); V-the total volume of the extract of the sample, (L); M-the mass of the sample, (kg).

### RNA Extraction and qRT-PCR Verification

Total RNA was extracted from 200 mg of tuberous roots using a RNAprep Pure Plant plus Kit (Tiangen, China). One-Step gDNA Removal and cDNA Synthesis SuperMix (TransGen, China) was used for first-strand cDNA synthesis. The qPCR reactions were performed in 10 μL volume in thermocycler (Thermo Fisher Scientific Inc., Göteborg, Sweden). The qPCR primers were shown in Supplementary Table 1. Reference gene of *MeActin* was used as an internal reference. All experiments of each gene were performed in triplicate per sample. The formula 2^–△△Ct^ method was used to calculate the relative gene expressions.

### Subcellular Localization Analysis

Full-length *MebHLH72* and *MebHLH114* were cloned into the transient expression vector pNC-Green-SubN and pNC-Green-SubC to generate *35S::GFP-MebHLH72* and *35S::GFP-MebHLH114* recombinant vector, *35S:: MebHLH72- GFP* and *35S:: MebHLH114 - GFP* recombinant vector, respectively (Yan et al., 2020). Then, *Agrobacterium tumefaciens* strain GV3101-psoup was cotransformed with the recombinant plasmid, and transiently expressed in *Nicotiana benthamiana* leaves. pNC-Green-SubN and pNC-Green-SubC were used as the positive control individually. The results were observed by laser scanning confocal microscope (TCS SP8, Leica) after 3 days.

### Virus-Induced Gene Silencing (VIGS) in Cassava and qRT-PCR Verification

For vector construction, 300 bp *MebHLH72, MebHLH114* and 300 bp *MeChlD* DNA fragments were cloned into pCsCMV-NC as described by Tuo et al., 2021. The primers are listed in Supplementary Table 2. pCsCMV-*ChlD300* as a positive control, which was a yellowing VIGS phenotype in the leaves. The recombinant plasmid co-transformed in *A. tumefaciens* strain GV3101-psoup, and cultured at 28 °C in chamber before resuspending in 10 mM MES, 10 mM MgCl_2_ and 20 mM acetosyringone. The preparations were injected into the back of cassava leaves using syringe, and grown in greenhouse (Yan et al., 2021). The expression level of the homolog genes that involved in CGs biosynthesis in *MebHLH72* and *MebHLH114* silenced lines were also detected, and the qPCR primers were shown in Supplementary Table 1.

### Statistical Analysis

Statistical Product and Service Solution program (version 20)(SPSS Inc., Chicago, IL, United States) was used for all statistical analysis. One-way ANOVA (Tukey) was conducted for the expression level and CGs in cassava tuberous roots and leaves comparisons, respectively.

## Results

### Identification and Phylogenetic Analysis of bHLH in *Manihot esculenta*

A number of 148 bHLH proteins were characterized from *M. esculenta* and named them from MebHLH1 to MebHLH148. 148 MebHLHs all contained the bHLH domain (PF0001) based on SMART tests and Pfam analysis. MebHLH proteins lengths are ranged from 89 (MebHLH54) to 735 (MebHLH74) amino acids, MW from 10.06 to 78.27 kDa, and p*I* from 4.36 to 10.32. Subcellular localization of MebHLHs was predicted by WoLF PSORT^10^. Among the 148 MebHLH proteins, four were predicted to be cytoplasmic proteins (MebHLH99, 133, 56 and 114), three were located in the chloroplast (MebHLH54, 30 and 44), one was mitochondrial protein (MebHLH124); one was plasma membrane protein (MebHLH66); one was extracellular protein (MebHLH87), and the rest of MebHLHs were localized in the nucleus. More detailed information including bHLH name, gene accession, chromosome locus, protein length, MW, p*I* of all identified MebHLH proteins were shown in Supplementary Table 3.

The bHLH domain sequences of MebHLH proteins and AtbHLH proteins were used to construct a phylogenetic tree by using neighbor-joining method. The results indicated that the 148 MebHLHs were classified into 19 groups according to the groups in *Arabidopsis* (Figure 1). Group 18 contained the highest bHLH members with 16 MebHLHs, followed by group 8 and group 9 with 14 MebHLHs. Group 12 had the least bHLH members with only one MebHLH. Additionally, a total of 40 MebHLHs members were clustered in group 2, group 6, group 9, group 12 and group 15, respectively, which play important roles in *Arabidopsis*. These results suggest that bHLH members in these groups of cassava could have the similar functions with those members in *Arabidopsis*.

### Chromosomal Locations, Duplications, and Synteny Analysis of the *MebHLH* Genes

To investigate the chromosomal distribution of the *MebHLH* genes, the DNA sequence of each *MebHLH* was obtained using blastn in cassava genome database. 148 *MebHLH* were mapped on 18 chromosomes (Figure 2). Distribution of *MebHLH* genes on 18 chromosomes was uneven. For instance, chromosome 06 and 14 contained the highest number (16) of *bHLH* genes, however, chromosome 11 contained the least number (3) of *bHLH* genes. Interestingly, many *MebHLH* genes were clustered in a short distance, such as the top of chromosome 14 and the bottom of chromosome 01 and chromosome 06.

To reveal the expansion mechanism of the *MebHLH* gene family, blastn and the CDS of all *MebHLH* genes was performed. Totally, 91 pairs (105 *MebHLH* genes) of segmental duplications were identified (Supplementary Table 4), seven pairs of tandem duplications (*MebHLH16*/*MebHLH17*, *MebHLH59*/*MebHLH60*, *MebHLH60*/*MebHLH61*, *MebHLH65*/*MebHLH66*, *MebHLH93*/*MebHLH94*, *MebHLH104*/*MebHLH105* and *MebHLH110*/*MebHLH111*) (Figure 3). Base on the Ka and Ks of each duplicated *MebHLH* gene pair, the Ka/Ks value of each gene pair was also calculated, gene pairs *MebHLH60/MebHLH61* (Ka/Ks > 1) may evolve under positive selection after duplication, other six gene pairs were less than 1, which indicated these genes had evolved under purifying selection.

To detect the synteny of *bHLH* genes, a collinearity analysis between *M. esculenta* and *A. thaliana* using MCScanX were performed. As a result, 116 paired collinearity relationships between 83 *MebHLH* and 78 *AtbHLH* genes were founded (Figure 4 and Supplementary Table 5).

### *MebHLH* Structures and Conserved Motifs

The exon/intron organization and conserved motifs were analyzed of all *MebHLH* genes (Figure 5 and Supplementary Figure 1). The exons number was ranged from 1 to 12, although *MebHLH1, MebHLH2, MebHLH3, MebHLH9, MebHLH13, MebHLH14, MebHLH16, MebHLH17, MebHLH37, MebHLH38, MebHLH42, MebHLH73, MebHLH83, MebHLH95, MebHLH106, MebHLH129, MebHLH136, MebHLH138, MebHLH141* contained only one exon, *MebHLH117* contained 11 exons, and *MebHLH53* contained 12 exons, which is the highest number of all genes. Additionally, *MebHLH* genes in same group had similar gene structure.

Ten conserved motifs were identified among the 148 MebHLH proteins. Notably, the members with high similarity in the same group shared a common motif composition. For instance, MebHLH24 and MebHLH100 were found to contain four motifs. It indicates that these two genes may have a similar function. MebHLH proteins (128/148, 86.49%) contain 2-4 conserved motifs. However, 11 MebHLH proteins (MebHLH129, MebHLH138, MebHLH37, MebHLH13, MebHLH1, MebHLH17, MebHLH83, MebHLH2, MebHLH3, MebHLH16, MebHLH136) contain eight motif and 5 MebHLH proteins (MebHLH32, MebHLH40, MebHLH11, MebHLH38 and MebHLH4) contain six motifs, 3 MebHLH proteins (MebHLH126, MebHLH14 and MebHLH9) contain five motifs, only MebHLH18 contain one seven motifs.

### *Cis*-Acting Regulatory Element Analysis

PlantCARE and PLACE database were used to characterize the *cis*-acting regulatory elements of each *MebHLH* promoter within 1,500 bp from transcription start site. Many kinds of *cis*-acting regulatory elements were existed in *MebHLH* (Supplementary Figure 2 and Supplementary Table 6). For instance, the common regulatory elements such as CAAT-box and TATA-box were presented in all 148 *MebHLH* genes. Furthermore, *cis*-acting regulatory elements related to hormone responses were also identified. Such as AuxRR-core and TGA-element, MYB which involved in abscisic acid (ABA) responsiveness; P-box and TATC-box, gibberellin responsiveness; CGTCA-motif and ERE, MeJA responsive elements. Interestingly, the promoter of some *MebHLH* genes also contained elements related to biotic and abiotic responses, including wounding (WUN-motif), cold (DRE and LTR), drought (MBS), pathogen defense (TC-rich repeat). In summary, *MebHLH* genes could be regulated by diverse hormone and environment.

### Expression Profile of *MebHLH*s and Cyanogenic Glucosides Changes in Response to Postharvest Physiological Deterioration

The expression levels of *MebHLHs* in tuberous roots during PPD were analyzed using RNA-seq data. The heatmap showed that compared with no PPD control, 117 (79.05%) *MebHLHs* were exhibited differences in their expression levels in response to PPD (Figure 6B). 43 *MebHLHs* showed significant differences in comparison with group II/I, 49 *MebHLHs* showed significant differences in comparison with group III/I, and 50 *MebHLHs* in comparison with group IV/I (Supplementary Table 7). Among them, 26 *MebHLHs* exhibited in all three comparison groups, 5 *MebHLHs* exhibited only in comparison with group II/I, 8 *MebHLHs* exhibited in comparison with group IV/I, and only *MebHL68* exhibited in comparison with group III/I (Figure 6C).

CGs biosynthesis was associated with PPD, therefore the CGs content including linamarin and lotaustralin in tuberous roots during PPD were also detected. It showed that linamarin content in sample C was significantly increased more than other samples, while lotaustralin was not detected in all samples (Table 1).

**TABLE 1:** Cyanogenic glucosides content of tuberous roots in four PPD samples and cassava leaves infected with pCsCMV-*bHLH72* and pCsCMV-*bHLH114*.

Content (μg/g)	Tuberous roots				Leaves			
	I	II	III	IV	SC9-Mock	pCSCMV-NC	pCSCMV*-bHLH72*	pCSCMV*-bHLH114*
**Linamarin**	33.90 ± 1.07B	50.66 ± 2.99B	157.38 ± 26.76A	39.96 ± 6.36B	263.52 ± 7.15B	329.83 ± 7.74A	126.89 ± 3.83C	85.80 ± 4.85D
**Lotaustralin**	n.d.	n.d.	n.d.	n.d.	119.89 ± 6.38A	112.93 ± 4.00A	116.26 ± 8.88A	104.25 ± 11.90A
**Total cyanogenic glycosides**	33.90 ± 1.07B	50.66 ± 2.99B	157.38 ± 26.76A	39.96 ± 6.36B	383.41 ± 8.92B	442.75 ± 11.12A	243.15 ± 11.60C	190.06 ± 8.19D

*One-way ANOVA (Tukey) was conducted for difference significance test, and different uppercase letters in the same row represented extremely significant difference (P < 0.01). n.d means not detectable in the sample.*

### Validation of *MebHLHs* Involved in Postharvest Physiological Deterioration

**FIGURE 7:**
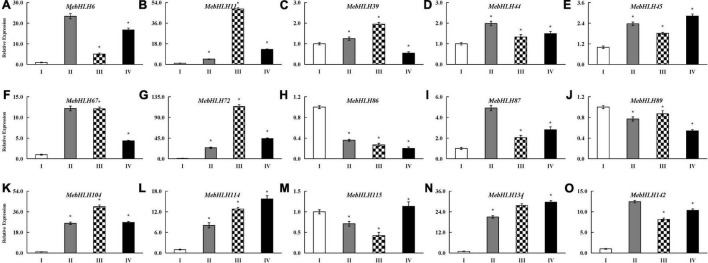
qRT-PCR results for *MebHLHs* under PPD process. qRT-PCR was used to analyze the expression profiles of 15 up-regulated *MebHLH* genes according to RNA-seq. PPD degrees I-IV were described in Figure 6A, **(A–O)** indicates the 15 *MebHLH* genes tested, respectively. The relative expression levels are normalized to *MeActin*. The data represent the mean of three biological replicates. The *x*-axis represents four materials from different PPD stage. The *y*-axis represents the relative expression levels of *MebHLHs*. All data are the Means ± SE of three independent experiments. Error bars indicate the standard deviation.

Based on the RNA-seq data, we identified 15 up-regulated genes during PPD, and quantitative real-time PCR (qRT-PCR) for the 15 candidate *MebHLHs* in four stages were performed. It showed that the expression of 12 *MebHLHs* were rise remarkably accompanied with PPD process, and they gradually decreased in the flowing stage (Figure 7), these results showed no significant difference with RNA-seq, where they were up-regulated compared with no PPD control. However, the expression levels of *MebHLH86*, *MebHLH89* in subfamily 16 and *MebHLH115* in subfamily 18 were less than no PPD control, it was not consistent with the RNA-seq results, that may be caused by different methods.

### *MebHLH72* and *MebHLH114* Is Necessary for Linamarin Biosynthesis in Cassava

**FIGURE 8:**
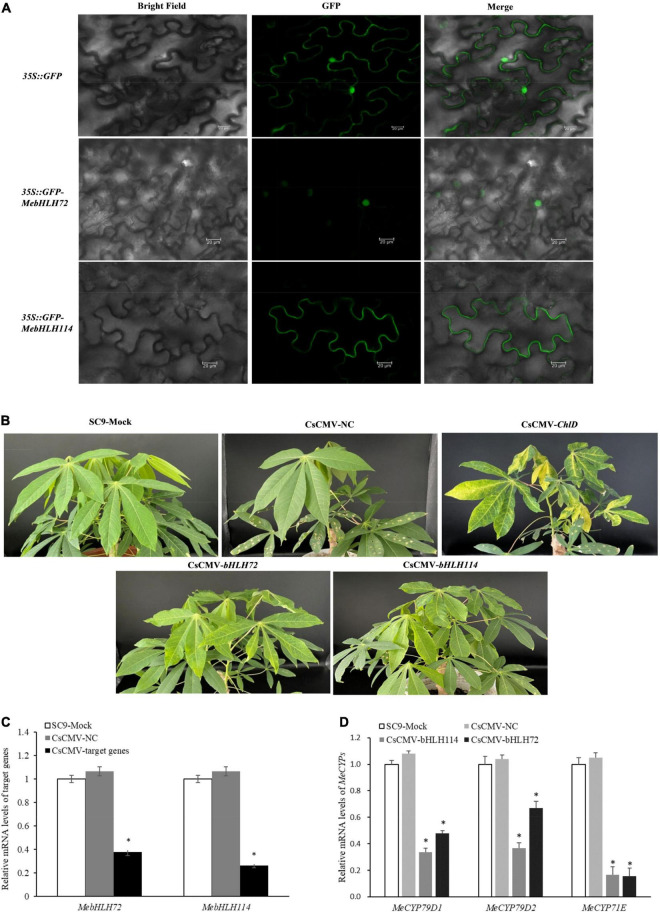
Subcellular localization and Silencing of *MebHLH72, MebHLH114* using the pCsCMV-*bHLH72* and pCsCMV-*bHLH114.* **(A)** Subcellular localization of MebHLH72 and MebHLH114 in tobacco leaves. Bars = 20 μm. **(B)** Silencing phenotypes in *M. esculenta* using the pCsCMV-*bHLH72* and pCsCMV-*bHLH114* at 40 dpi. SC9-Mock means no infected plant, pCsCMV-NC means infected with non-target control CsCMV-NC. **(C)** qRT-PCR analyses of *MebHLH72* and *MebHLH114* mRNA expression in *M. esculenta* infected with pCsCMV-*bHLH72* and pCsCMV-*bHLH114*. The no infected plant was used as control. Three independent experiments were performed and each included six plants per treatment group. Error bars indicate the standard deviation. **(D)** qRT-PCR analyses of *MeCYP79D1, MeCYP79D2 and MeCYP71E* mRNA expression in *M. esculenta* infected with pCsCMV-*bHLH72* and pCsCMV-*bHLH114*. The no infected plant was used as the control. Three independent experiments were performed and each included six plants per treatment group. Error bars indicate the standard deviation.

We investigated the subcellular localization of MebHLH72 and MebHLH114 proteins *in planta*. Consistent with a previous study, GFP-fused MebHLH72 protein was localized in nucleus, and GFP-fused MebHLH114 protein was localized in cytoplasm even in tobacco leaves (Figure 8A), indicating their similar localization with prediction in Supplementary Table 3. To study the *in vivo* roles of *MebHLHs* in cassava CGs biosynthesis, two gene-silenced cassava lines were constructed (*pCsCMV-bHLH72* where *MebHLH72* was silenced, and *pCsCMV-bHLH114* where *MebHLH114* was silenced), the phenotypes between *MebHLHs*-silenced lines and control plants were shown in Figure 8B. When the transcript levels of *MebHLH72* and *MebHLH114* were evaluated in *MebHLH*-silenced lines, the transcripts level of targeted *MebHLHs* were drastically reduced compared with pCsCMV-NC and Mock (Figure 8C). We compared the CGs content including linamarin and lotaustralin of *MebHLHs*-silenced lines in leaves with that of control plants, the linamarin content was significantly decreased in the leaves of both *MebHLH72*- and *MebHLH114*-silenced. However, the lotaustralin content was no significantly differences in leaves of *MebHLH*-silenced lines compare with that of control plants (Table 1). In addition, the expression level of the homolog genes that involved in CGs biosynthesis, *MeCYP79D1, MeCYP79D2 and MeCYP71E* were also detected in *MebHLH72* and *MebHLH114* silenced lines (Figure 8D). And the relative expression of all three genes was significantly reduced in two silenced lines. Therefore, *MebHLH72* and *MebHLH114* may be candidate genes involved in CGs biosynthesis.

## Discussion

bHLHs, one of the largest transcription factor families, have been investigated in various plants species. In this study, 148 *bHLH* gene family members were identified in cassava genome, 136 bHLH proteins in *Arabidopsis* were used to construct phylogenetic tree, As an illustration of the size of the bHLHs family in plants, 164 bHLHs have been found in *Arabidopsis* (Carretero-Paulet et al., 2010), while there are 180 bHLHs in Rice (Xiong et al., 2005), 225 bHLHs in Wheat (Guo and Wang, 2017), 190 bHLHs in Tobacco (Rushton et al., 2008), 191 bHLHs in Grapes (Jaillon et al., 2007), 102 bHLHs in Walnut (Zhao et al., 2021), 159 bHLHs in Tomato (Sun et al., 2015). Figure 2 showed that 148 *MebHLH* genes were unevenly distributed on 18 chromosomes, and some *MebHLH* genes were clustered on top or bottom of chromosomes. Based on phylogenetic analysis, 148 bHLHs were classified into 19 subfamilies according to the subfamilies in *Arabidopsis* (Figure 1; Toledo-Ortiz et al., 2003), which was probably due to the methods and sequences were adopted (Carretero-Paulet et al., 2010; Song et al., 2013).

Gene duplication plays an essential role in genome amplification, species evolution and gene family evolution (Lynch and Conery, 2000). Gene duplication is including three main types: whole genome duplication, tandem duplication, and segmental duplication (Yu et al., 2005). In this study, 91 pairs of segmental duplications were identified (Supplementary Table 4), 7 pairs of tandem duplications in cassava (Figure 3). Tandem duplicated genes were less common relatively, this was consistent with results in barley and tobacco (Ke et al., 2020; Bano et al., 2021). These results implied that segmental duplication is the main style in cassava genome. Duplicated genes may exhibit functional diversity, and the mechanisms could be identified by sequencing. Only one pair of tandem duplicated genes (*MebHLH60/MebHLH61*) was uncovered in cassava with Ka/Ks > 1, which suggest that positive selection was occurred on *MebHLH60/MebHLH61*.

*Cis-*acting elements were analyzed in this study and a large number of hormone response elements were founded (AuxRR-core and TGA-element, ABRE, CGTCA-motif, TGA-element), stress response elements (WUN-motif, LTR, ARE, TC-rich repeat and MBS) (Supplementary Figure 2 and Supplementary Table 6). It implied that *MebHLH* genes may be involved in various stress responses and could be induced by many hormones. For example, exogenous MeJA could enhance the activity of *bHLH* promoters (Chen et al., 2019; Yu Z. et al., 2021), and then participating in the regulation of plant physiological processes.

As one of the most numerous TFs in plant, the bHLH family has many members and various functions. bHLHs can regulate plant resistances to diverse stresses including cold, drought, salt and wound (Guo et al., 2021; Qian et al., 2021). Postharvest physiological deterioration is one of the major constraints to commercial production and utilization of cassava (Saravanan et al., 2016), it was induced by wounding, which initiates from wound sites and triggers an oxidative burst of the superoxide radical (O_2_^–^) within 15 min, and further production of ROS (Zidenga et al., 2012). This was supported by earlier transcriptome data, 20% of the genes were involved in ROS turnover during PPD process (Reilly et al., 2007), and overexpression of superoxide dismutase (SOD) and catalase (CAT) would delayed PPD occurrence of cassava tuberous roots (Xu et al., 2013). As well, many previous reports showed that flavonoids synthesized in plant could effectively eliminate ROS to enhance the tolerance in adverse environment (Wang et al., 2016; Qin et al., 2020). Interestingly, some bHLHs were confirmed to be associated with flavonoids synthesis (Zhao et al., 2019; Aslam et al., 2020) and belongs to subfamilies 2 (Guan et al., 2013; Geng and Liu, 2018; Geng et al., 2019; Waseem et al., 2019; Zhao R. et al., 2020). Especially, experimental data showed that bHLHs bound to the promoter of antioxidant genes such as *POD, SOD* through E-box or G-box (Liu et al., 2013; Geng and Liu, 2018). Most *bHLHs* in subfamilies 1, 2, 5 and 15 could bind the E-box and the G-box (Yao et al., 2018; Xi et al., 2021). In our study, the majority of PPD responsive *MebHLHs* are from subfamilies 2, 5, 10, 15, 16 and 18. And the expression profile of some genes related to flavonoids synthesis and ROS scavenging were significantly difference in the RNA-seq data, such as *MeANR, MeF3H, MeANS* associated with flavonoids synthesis, and *MePOD, MeCAT, MeAPX* involved in ROS scavenging (Supplementary Figure 3A). Furthermore, all these genes have G-box in their promoters (Supplementary Figure 3B). qRT-PCR results of 15 up-regulated *MebHLHs* during PPD showed that *MebHLH114* in subfamilies 2, *MebHLH67 and MebHLH104* in subfamilies 5, *MebHLH72 and MebHLH134* in subfamilies 10 were up-regulated compared with no PPD control, it was consistent with the RNA-seq results. Given all of that, it is reasonable to hypothesize that bHLHs in subfamilies 2, 5 and 15 could bind to E-box or G-box of target genes related with ROS scavenging to further regulate the PPD process. However, these hypotheses still need to be verified.

CGs play an important role in plant defense response, and its correlation with PPD has been confirmed (Reilly et al., 2007). The linamarin content increased in cassava tuberous roots of sample III (Table 1), and linamarase protein were accumulated at four days after PPD initiation (Owiti et al., 2011). The increase of linamarin content could be due to the up-regulation of the genes *CYP79D1, CYP79D2*, and *CYP71E* involved in CGs biosynthesis (Jørgensen et al., 2011). LjbHLH7 can directly activate the expression of *CYP79D3* gene by binding to the G-box sequence of its promoter (Chen et al., 2022). In our study, the linamarin content significantly decreased in the leaves when *MebHLH72* and *MebHLH114* were silenced individually. The relative expression of *CYP79D1, CYP79D2*, and *CYP71E* were decreased in *MebHLH72* and *MebHLH114* silenced lines (Figure 8D), and all three genes have two G-box in their promoters (Supplementary Figure 3C). It means that *MebHLH72* and *MebHLH114* were involved in CGs biosynthesis, probably through combing with G-box of gene promoter which participates in CGs biosynthesis. As transcript factors, most of them were localized in nucleus, however, MebHLH114 (fusion with n/c-*GFP*) was localized in cytoplasm in this study (Figure 8A and Supplementary Figure 4). Previous report indicated that subcellular localization of AtbHLH039 was also localized at the cell periphery and only a rather weak presence in the nucleus (Trofimov et al., 2019). Recent research suggested that the transport factors will be altered the subcellular localization when environment changed or post translational modification occurred (Wang et al., 2021). Therefore, we inferred that the subcellular localization of MebHLH114 may be changed during the PPD process.

## Conclusion

In this study, a genome-wide analysis of *MebHLH* gene family was characterized with particular focus on their response to cassava PPD and CGs biosynthesis. A total of 148 *MebHLH* genes were identified and characterized. The chromosomal distribution, gene structure, gene duplication, *cis*-elements, covered motif and expression profiles of *MebHLHs* were analyzed. Moreover, transcriptome data analyses unveiled that 65 *MebHLH* genes may play a crucial roles in PPD of tuberous root. During PPD, CGs biosynthesis was increased, linamarin content was significantly decreased in the leaves of cassava plants with silenced *MebHLH72* and *MebHLH114*. Overall, the results will provide a new insight for cassava engineering programs, including improved PPD tolerance and decreased CGs content.

## Data Availability Statement

The datasets presented in this study can be found in online repositories. The names of the repository and accession numbers can be found below: https://www.ncbi.nlm.nih.gov/, PRJNA841274.

## Author Contributions

FA: conceptualization and original draft preparation. XX: software, review and editing, and visualization. TC: methodology and project administration. JX: validation form analysis. XL: data curation. WO: investigation. KL: resources. SC and JC: modification and supervision. WO, KL, and SC: funding acquisition. All authors have agreed to the published version of the manuscript.

## Conflict of Interest

The authors declare that the research was conducted in the absence of any commercial or financial relationships that could be construed as a potential conflict of interest.

## Publisher’s Note

All claims expressed in this article are solely those of the authors and do not necessarily represent those of their affiliated organizations, or those of the publisher, the editors and the reviewers. Any product that may be evaluated in this article, or claim that may be made by its manufacturer, is not guaranteed or endorsed by the publisher.

## Footnotes

1
https://phytozome.jgi.doe.gov/

2
http://pfam.sanger.ac.uk/

3
http://smart.embl-heidelberg.de/

4
https://www.expasy.org/

5
http://gsds.gao-lab.org/

6
http://meme-suite.org/tools/meme

7
http://bioinformatics.psb.ugent.be/webtools/plantcare/html/

8
https://www.dna.affrc.go.jp/PLACE/

9
https://www.megasoftware.net/

10
https://wolfpsort.hgc.jp/
