# Supplementary material for: Systematic Analysis of bHLH Transcription Factors in Cassava Uncovers Their Roles in Postharvest Physiological Deterioration and Cyanogenic Glycosides Biosynthesis
Source: Front Plant Sci. 2022 Jun 16;13:901128. doi: 10.3389/fpls.2022.901128 (PMC9249602; doi:10.3389/fpls.2022.901128)
Supplement: Supplementary file 8 [file Image_1.PDF]

```
                            *        20         *        40         *
Mebhlh117  : -RSKHSATEQRRRSKINDRFQKLRELIPHG---DQ--KRDKASFLLEVIEYIQFLQ : 50
Mebhlh122  : -----SIAARERRRKITEKTQELGKLIPGG---SK---MNTAEMLQAASQYVKFLQ : 45
Mebhlh112  : ------MAERRRRKKLNDRLYMLRSVVPK-----IS-KMDRASILGDAIEYLKEL- : 43
Mebhlh116  : ----HNQSERKRRDRINQKMKALQKLVPNA---SK---TDKASMLDEVIEYLKQLQ : 46
Mebhlh115  : ---SHSLAERARREKISERMKILQDLVPGC---NK--VIGKALVLDEIINYIQSLQ : 48
Mebhlh111  : -KLLHRDIERQRRQEMTTLYASLRSLLPLE---YIKGKRSISDHMNEAVNYIKHLK : 52
Mebhlh123  : ------VSERNRRKKLNERLFALRAVVPN-----IS-KMDKASIIKDAIDYIQELH : 44
Mebhlh120  : --KSHSEAERRRRERINNHLAKLRSLL-PS---TTK--TDKASLLAEVIQHVKEL- : 47
Mebhlh110  : KKIMHRDFERRRRQDMTTLYTSLRNLLPLE---YIKGKRAISDQIHQAEKYIKDLQ : 53
Mebhlh108  : ----RSIAERVRRTRISERMRKLQDLVPNM---DK--QTNTADMLDLAVDYIKDLQ : 47
Mebhlh121  : ----HSIAERLRRERIAERMKALQELVPNA---NK--TD-KASMLDEIIDYVKFLQ : 46
Mebhlh109  : QKISHITVERNRRKQMNEHLSALRSLMPCF---YVK-RGDQASIIGGVVDYINELQ : 52
Mebhlh118  : -RSKHSVTEQRRRSKINERFQILRDLIPHS---DQ--KRDTASFLLEVIEYVQFLQ : 50
Mebhlh114  : ----RKTVERNRRIHMKGLCFKLASLIPSHHFKHSKDMLSQQGQLDHAAAYIKHLK : 52
Mebhlh113  : ---SHSLAERVRREKISERMKILQRLVPGC---DK--VTGKALMLDEIINYVQSLQ : 48
Mebhlh119  : --NSHSLAERVRREKISERMKFLQDLVPGC---SK--VTGKAVMLDEIINYVQSLQ : 49
Mebhlh69   : --KSHSQAEKRRRDRINAQLGILRKLI-PK---SDK--MDKAALLGSVIDQVRDL- : 47
Mebhlh64   : -----SIAERVRRTRISERMRKLQDLFPNM---DK--QTNTADMLDLAVEYIKDLQ : 46
Mebhlh67   : --QDHIIAERKRREKLSQRFIALSAIVPGL---KK---MDKASVLGDAIKYLKQLQ : 48
Mebhlh68   : ---------RKRRERINERLRILQNLVPNG---TK---VDISTMLEEAVHYVKFLQ : 41
Mebhlh66   : ----------ERRREMQDKYQTLRNLIPNP---NPT-KYDRASVIGDAVEYVKEL- : 41
Mebhlh65   : --TKHFATERQRRQHLTDKYQALKDLVPN-----PT-KNDRASVVGDAINYIKEL- : 47
Mebhlh52   : -RSKHSVTEQRRRSKINERFQILRDLIPHS---DQ--KRDTASFLLEVIEYVQYLQ : 50
Mebhlh53   : -RSKHSATEQRRRSKINDRFQMLRELIPHV---DQ--KRDKASFLLEVIEYIQFLQ : 50
Mebhlh59   : KKKLHRDIERQRRQEMATLYASLRSLLPLQ---YIKGKRSISDHMNEAVNYINHLQ : 53
Mebhlh51   : --KSHSEAERRRRERINNHLAKLRSLL-PS---TTK--TDKASLLAEVIQHVKEL- : 47
Mebhlh58   : ------MAERRRRKKLNDRLYMLRSVVPK-----IS-KMDRASILGDAIEYLKEL- : 43
Mebhlh62   : QRMSHITVERNRRKQMNEHLSVLRSLMPCF---YVK-RGDQASIIGGVVDYINELQ : 52
Mebhlh56   : ----RKTVERNRRIHMKGLCFKLASLIPSHRFKHSKELLSQQDQLDHAAAYIKHL- : 51
Mebhlh50   : ---SHSLAERVRREKINEKLKNLQNLVPGC---YK--TMGMAVMLDVIINYVQSLQ : 48
Mebhlh55   : ---SHSLAERARREKISERMKMLQDLVPGC---NK--VIGKALVLDEIINYIQSLQ : 48
Mebhlh61   : ----RKEIERQRRQQMSTLCASLRSLLPLE---SLKGKRAISDHINEAAKYIKDLQ : 49
Mebhlh63   : ----RSIAERVRRTRISERMRKLQDLVPNM---DK--QTNTADMLDLAVDYIKDLQ : 47
Mebhlh60   : KRNVHRDIEKRRRQEMTTLYTSLRNLLPLE---YIKGKRAMSDHIHEAVKYINDLQ : 53
Mebhlh48   : -------SERNRRKKLNDRLFALRAVVPN-----IS-RMDKASIIKDAIDYIQELH : 43
Mebhlh49   : -----SIAARERRRKITEMTQELGKLVPGG---NK---MNTAEMLQAASKYVKFMQ : 45
```

```
Mebhlh54  : -------------DDINELIFKLQALLPQLNQ-RQASRASASRVLKETCRYIRKLR : 42
Mebhlh57  : ---SHSLAERVRREKISERMKILQQLVPGC---DK--VTGKALMLDEIINYVQSLQ : 48
Mebhlh142 : ---VHILTERERRKKMRNMFTSLHALLPQL---PAK--ADKSTIVDEAIKYIKNLQ : 48
Mebhlh144 : ---------RKRRERINERLRILQNLVPNG---TK---VDISTMLEEAVHYVKFLQ : 41
Mebhlh146 : -----LMAERRRRKRLNDRLSMLRSIVPK-----IS-KMDRTSILGDTIDYVKEL- : 44
Mebhlh148 : QRMTHIAVERNRRKQMNEYLAVLRSLMPPS---YVQ-RGDQASIIGGAINFVKELE : 52
Mebhlh143 : ---VHNLSEKRRRSRINEKMKALQNLIPNS---NK---TDKASMLDEAIEYLKQLQ : 47
Mebhlh145 : --NSHSLAERVRREKISERMRLLQELVPGC---NK--ITGKAVMLDEIINYVQSLQ : 49
Mebhlh147 : -CKSHKEAERRRRQRINAHLSTLRTLL-PS---TTK--TDKASLLAEVVHHVKEL- : 48
Mebhlh141 : ---------RHRRERISERIRILQRLVPGG---TK---MDTASMLDEAIHYVKFL- : 40
Mebhlh26  : ----HVISERRRREKINETFEALRKLLPPV---AK---KDKASVLNRTREYLTSL- : 45
Mebhlh27  : ---SKASREKLRRDRLNDKFVELGSILEPG---RPP-KTDKAAILIDAVRMVSQL- : 48
Mebhlh29  : --ATKACREKLRRERLNDRFQDLSSVLEPG---RPA-RTDKTAILADAIRVLTRL- : 49
Mebhlh30  : ---SHITVERNRRRQMNEHLKVLRSLTPCF---YIK-RGDQASIIAGVVEFIKELH : 49
Mebhlh28  : QRMTHIAVERNRRKQMNEHLRVLRSLMPGS---YVQ-RGDQASIIGGAIEFVRELE : 52
Mebhlh40  : --ANHVLAERRRREKLNERFIILRSLVPFV---TK---MDKASILGDTIEYVKQLR : 48
Mebhlh46  : ---SHSLAERVRREKISERMKFLQDLVPGC---NK--VTGKAVMLDEIINYVQSLQ : 48
Mebhlh44  : ---------RKRRERINERLRILQNLVPNG---TK---VDISTMLEEAVHYVKFLQ : 41
Mebhlh41  : ---SHSLAERARREKISERMKILQDLVPGC---NK--VIGKALVLDEIINYIQSLQ : 48
Mebhlh37  : ---NHVEAERQRREKLNHRFYALRAVVPNV---SR---MDKASLLSDAVCYINEL- : 46
Mebhlh38  : ---NHVEAERQRREKLNQRFYALRAVVPNI---SK---MDKASLLGDAIAYINELQ : 47
Mebhlh47  : RKIQKADREKLRRDRLNEHFIELGNKLDPD---RP--KNDKATILADTIQLLKEL- : 50
Mebhlh36  : QRMTHIAVERNRRRQMNDHLNSLRSLMPPS---YVQ-RGDQASIIGGAIDFVKELE : 52
Mebhlh45  : -----LMAERRRRKRLNDRLSMLRSIVPK-----IS-KMDRTSILGDTIDYVKEL- : 44
Mebhlh43  : ----HILTERERRKKMRNMFSNLHALLPEL---PAK--ADKSSIIDEAVKYIKILQ : 47
Mebhlh35  : RREVHKQSERKRRDKITKKMRALQALLPNS---TK---VDKVSVLDNAIEYLKTLQ : 50
Mebhlh42  : ---------RHRRERISEKIRILQRLVPGG---TK---MDTASMLDEAIHYVKFL- : 40
Mebhlh39  : ----HNQSERKRREKINQRMKTLQKLVPNS---SK---TDKASMLDEVIEYLKQLQ : 46
Mebhlh5   : -RSKHSETEQRRRSKINERFQVLRDLIPQN---DQ--KRDKASFLLEVIEYIQFLQ : 50
Mebhlh15  : ----HNQSERKRRDKINQRMKTLQKLVPNS---SK---TDKASMLDEVIEYLKQLQ : 46
Mebhlh8   : ---SHSLAERARREKISERMKILQDLVPGC---NK--VIGKALVLDEIINYIQSLQ : 48
Mebhlh11  : --ANHVLAERRRREKLNERFIILRSLVPFV---TK---MDKASILGDTIEYVKQLR : 48
Mebhlh13  : ---NHVEAERQRREKLNHRFYALRAVVPNV---SR---MDKASLLSDAVSYIKEL- : 46
Mebhlh12  : QRMTHIAVERNRRRQMNDHLNSLRSLMPSS---YVE-RGDQASIVGGAIDFVRQLE : 52
Mebhlh9   : ---NHVEAERQRREKLNQRFYALRAVVPNI---SK---MDKASLLGDAITYITDLQ : 47
Mebhlh2   : ---NHVEAERQRRERLNHRFYALRSVVPNV---SK---MDKASLLADAVTYIKEL- : 46
Mebhlh6   : ---SHSIAERVRREKINNKLRCLQDLVPGC---HK--SMGMAVMLEEIINYVHSLQ : 48
```

```
Mebhlh3   : ---NHVEAERQRRERLNHRFYALRSVVPNV---SK---MDKASLLADAVTYIKEL- : 46
Mebhlh1   : ---HHVEAERKRRERLNHRFYALRSVVPNV---SK---MDKASLLADAVTYIKDL- : 46
Mebhlh14  : ---NHVEAERQRREKLNQRFYALRAVVPNI---SK---MDKASLLGDAISYISELQ : 47
Mebhlh10  : ----HSIAERLRRERIAERIRALQELVPSV---NK--TD-RAAMLDEIVDYVKFL- : 45
Mebhlh4   : --ANHVLVERKRRERLKENFMILKSSFYSI---KR---ADKVSVLDETIEYMQELE : 48
Mebhlh7   : RKVQKADREKLRRDRLNEHFLELGNTLDPD---RP--KNDKATILTDTIQVLKDL- : 50
Mebhlh72  : ---NHKEAEKRRRERINSHLDKLRNLL-PC---NSK--TDKASLLAKVVQRVREL- : 46
Mebhlh74  : ---VHNLSERRRRDRINEKMRALQELIPNC---NK---VDKASMLDEAIEYLKTLQ : 47
Mebhlh75  : QRMTHIAVERNRRKQMNEYLSILRSLMPES---YVQ-RGDQASIIGGAINFVKELE : 52
Mebhlh73  : ---------RERRHRISDRFKILQSLVPGG---SK---MDTVSMLEEAINYVKFL- : 40
Mebhlh70  : -----SIAERVRRTRISERMRKLQELVPNM---DK--QTNTADMLDLAVEYIKDLQ : 46
Mebhlh76  : ----HSIAERLRRERIAERMKALQELVPTA---NK--TD-RAAMLDEIVDYVKFL- : 45
Mebhlh71  : KKLNHNASERHRRKKMNSLYSSLRSLLPAA---DQMKKLSIAATVSGVLKYIPELQ : 53
Mebhlh140 : ---SHSEAERRRRERINAHLATLRGLI-PC---AEK--MDKATLLAEVINQVKEL- : 46
Mebhlh139 : ---KNLHAERRRRQKLGDSLLALRSLVPI-----IT-NMNKAAIIVDAITYIKELQ : 47
Mebhlh138 : ---NHVEAERQRREKLNQRFYSLRAVVPNV---SK---MDKASLLGDAIAYIKELR : 47
Mebhlh90  : -----LISERRRRGRMKEKLYALRSLVPN-----IT-KMDKASIIGDAVQYVQELQ : 45
Mebhlh89  : ----RSIAERERRTRISGRLKKLQELVPNM---DK--QTSYSDMLDLAVQHIKGLQ : 47
Mebhlh87  : ---------RQRREKISERIRVLQRLVPGG---SK---MDTASMLDEAANYLKFL- : 40
Mebhlh88  : ---SHSLAERVRREKISERMKYLQDLVPGC---NK--ITGKAGMLDEIINYVQSLQ : 48
Mebhlh83  : ---NHVEAERQRRERLNRRFYALRSVVPNV---SK---MDKASLLADAVTYINEL- : 46
Mebhlh81  : -------TERERRKKMRNMFANLHALLPQL---PPK--ADKSTIVDEAISYIKTLQ : 44
Mebhlh85  : --TKHFATERQRRQHLNDKYKALRDLVPN-----PT-KNDRASVVGDAIEYIKEL- : 47
Mebhlh84  : --QDHTIAERKRREKLSQRFIALSAIVPGL---KK---MDKASVLGDAIKYLKQLQ : 48
Mebhlh86  : -----SIAERVRRMRISERMRKLQDLFPNM---DK--QTNTADKLDFAVEYIKDLQ : 46
Mebhlh82  : ---------RKRRERINERLKILQNLVPNG---TK---VDISTMLEEAVNYVKFLQ : 41
Mebhlh105 : ----HVLAERKRREKLSQRFISLSAVVPGL---KK---MDKASVLGDAIKYLKHLQ : 46
Mebhlh101 : ---VHNLSERRRRDRINEKMKALQELIPRC---NK---SDKASMLDEAIEYLKSLQ : 47
Mebhlh107 : RKTERKVIEKIRRNQMKTLFSNLYSLLPKQ---SSQEALPLTDQIDEAINYIKT-- : 51
Mebhlh102 : ---SHSLAERVRREKISERMKLLQDLVPGC---NK--VTGKALMLDEIINYVQSLQ : 48
Mebhlh103 : -----LMAERRRRKRLNDRLSMLRSIVPK-----IS-KMDRTSILGDTIDYVKEL- : 44
Mebhlh106 : ---------RHRRERISERIRILQRLVPGG---TK---MDTASMLDEAIHYVKFL- : 40
Mebhlh104 : ---DHILAERKRREKLSQRFIALSAIVPGL---KK---MDKASVLGDAIKHVKQLQ : 47
Mebhlh79  : QRMTHIAVERNRRKQMNEYLSVLRSLMPDS---YVQ-RGDQASIIGGAINFVKELE : 52
Mebhlh80  : ---VHNLSERRRRDRINEKMRALQELIPNC---NK---VDKASMLDEAIEYLKTLQ : 47
Mebhlh77  : -----SIAERVRRTRISERMRKLQELVPNM---DKQ-QTNTADMLDLAVVYIKDLQ : 47
Mebhlh78  : ----HSIAERLRRERIAERMKALQELVPTA---NK--SD-RAAMLDEIVDYVKFL- : 45
```

```
Mebhlh32  : -------AERRRRKRLNGRLYDLRALVPK-----IS-NLNKAAILGDAIEFVKELQ : 43
Mebhlh34  : ------MAERRRRKRLNDRLYMLRSVVPK-----IS-KMDRASILGDAIDYLKEL- : 43
Mebhlh31  : ----RSIAERERRTRISGRLKKLQELVPNM---DK--QTSYADMLDLAVQHIKVLQ : 47
Mebhlh33  : ---SHSLAERVRREKISERMKYLQDLVPGC---NK--ITGKAGMLDEIINYVQSLQ : 48
Mebhlh16  : ---NHVEAERQRRERLNHRFYALRSVVPNV---SK---MDKASLLADAVTYIKEL- : 46
Mebhlh18  : --ANHALVERKRREKLNERFMILKSIVPSI---NK---VDKVSILDETIEYLQELE : 48
Mebhlh20  : ---SHSIAERVRREKINNKLRCLQDLVPGC---HK--SMGMAVMLEEIINYVHSLQ : 48
Mebhlh21  : ------IAAKNRRERISERLKVLQELVPNG---SK---VDLVTMLEKAISYVKFLQ : 44
Mebhlh23  : -CKSHKEAERRRRQRINAHLSTLRTLL-PN---ATK--TDKASLLAEVVHHVREL- : 48
Mebhlh17  : ---NHVEAERQRRERLNHRFYALRSVVPNV---SK---MDKASLLADAVTYIQGL- : 46
Mebhlh25  : ----RSIAERVRRTRISDRIRKLQELVPNM---DK--QTNTADMLDEAVEYVKFLQ : 47
Mebhlh22  : ---SHSLAERARREKISKKMKCLQELVPGC---NK--ITGRAGLLDEIINYIQSLQ : 48
Mebhlh19  : -RSKHSETEQRRRSKINERFQILRDLIPQN---DQ--KRDKASFLLEVIEYIQFLQ : 50
Mebhlh24  : QRMTHIAVERNRRKQMNEYLAVLRSLMPPS---YVQ-RGDQASIVGGAINFVKELE : 52
Mebhlh133 : -RTDKKTIERNRRNQMKALCSKLNSLLPHQ---SSRKPMSLSDQLDQAAKYIKKLQ : 52
Mebhlh132 : ---SKACREKMRRDRINDRFLELSALLDSG---RPP-KVDKSAILADALKVVNQL- : 48
Mebhlh136 : ---NHVEAERQRRERLNHRFYALRSVVPNV---SK---MDKASLLADAVTYIKEL- : 46
Mebhlh134 : ---SHSEAEKRRRERINAHLASLRGLV-PC---GEK-KMDKATLLAEVINQVKEL- : 47
Mebhlh131 : ---KNLLAERRRRKKLSDRLLALRSLVPT-----IT-NMNKATIIEDAITYIQELQ : 47
Mebhlh135 : ----HSIAERLRREKIAERMKNLQELVPNS---SK--ID-KASMLDEIIEYVKFLQ : 46
Mebhlh137 : ---HHMISERRRREKINESFEALRKLLPPE---AK---KDKASVLTRTRDYLTSL- : 46
Mebhlh91  : ---KNLVTERNRRNRMKDGLFTLRALVPK-----IS-KMNKAAILGDAIDYIGELQ : 47
Mebhlh97  : --QEHILAERKRREKLSQRFIALSAIVPGL---KK---MDKASVLGDAISYVKQLQ : 48
Mebhlh92  : ---VHNLSERRRRDRINEKMRALQELIPRC---NK---SDKASMLDEAIEYLKSLQ : 47
Mebhlh98  : -----LMAERRRRKRLNDRLSMLRSIVPK-----IS-KMDRTSILGDTIDYMKEL- : 44
Mebhlh100 : QRMTHITVERNRRKQMNDYLAVLRSMMPPS---YVQ-RGDQASIVGGAINFVKELE : 52
Mebhlh96  : ---VHNLSEKRRRSRINEKMKALQNLIPNS---NK---TDKASMLDEAIEYLKQLQ : 47
Mebhlh99  : --QKHKEAERKRRIRINDQYANLRTVL-PN---LIK--RNKASVLAETIQRVKELE : 48
Mebhlh93  : -KTERKVIEKNRRNQMKTLFSNLNSLLPKQ---SSKEALPLPDQVDEAINYIKSLE : 52
Mebhlh95  : ---------RHRRERISERIRILQRLVPGG---TK---MDTASMLDEAIHYVKFL- : 40
Mebhlh94  : -KTERKVIEKNRRNQMKTLFSNLNSLLPKQ---SSKEALPLPDQVDEAINYIKSLE : 52
Mebhlh129 : ---NHVEAERQRREKLNQRFYALRAVVPNV---SK---MDKASLLGDAIAYIDEL- : 46
Mebhlh128 : ---VHNLSEKRRRSRINEKIKALQNLIPNS---NK---TDKASMLDEAIEYLKQLQ : 47
Mebhlh130 : QRMTHIAVERNRRKQMNEYLAILRSLMPES---YVQ-RGDQASIVGGAIEFVKELE : 52
Mebhlh125 : --RTKACREKLRREKLNDRFQDLSSVLEPG---RPA-RIDKPAILDDAVRVLNQL- : 49
Mebhlh126 : QRMTHIAVERNRRKQMNEHLRVLRSLMPGS---YVQ-RGDQASIIGGAIEFVRELE : 52
Mebhlh127 : ---SKACREKLRRDRLNDKFLELGSILEPG---RPP-KTDKAAILIDAVRMVTQL- : 48
```

```
Mebhlh124 : ---RHISVERNRRRQMNEHLKVLRSLTPCF---YIK-RGDQASIIGGVIEFIKELH : 49
                                e  rr  6      L    p              6      6
```

Supplementary Figure 1 | Analysis of the conserved domain regions in the 148 MebHLHs.
