# Supplementary figures and images for: Systematic Analysis of bHLH Transcription Factors in Cassava Uncovers Their Roles in Postharvest Physiological Deterioration and Cyanogenic Glycosides Biosynthesis
Source: Front Plant Sci. 2022 Jun 16;13:901128. doi: 10.3389/fpls.2022.901128 (PMC9249602; doi:10.3389/fpls.2022.901128)

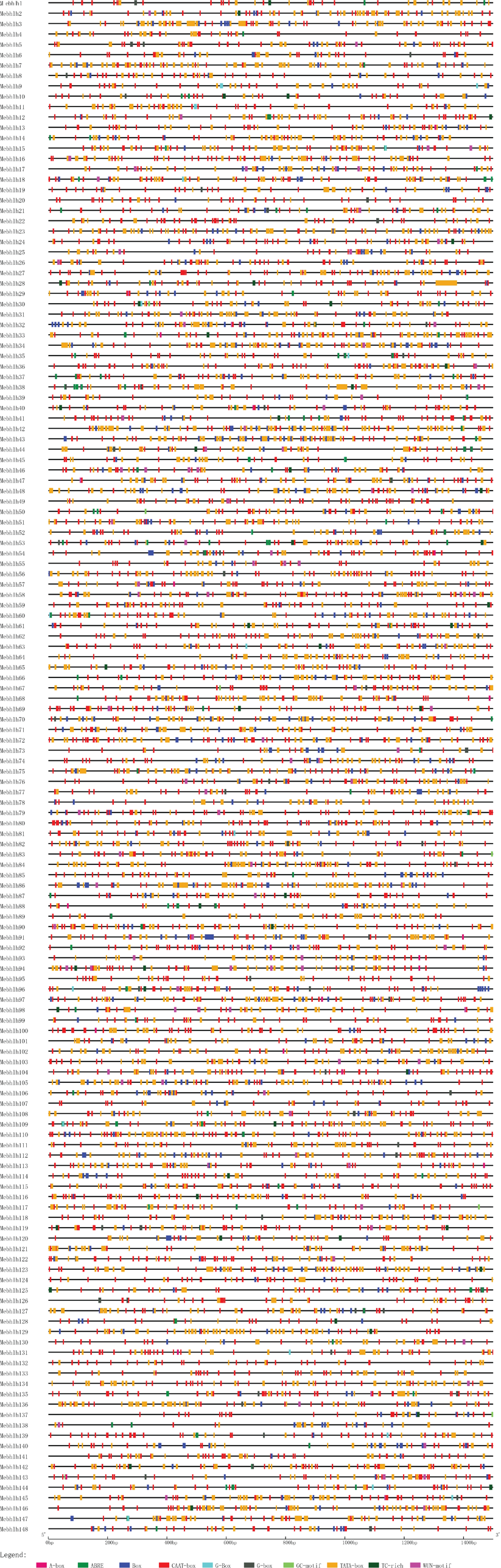

Supplement: Supplementary file 9 [file Image_2.TIFF]

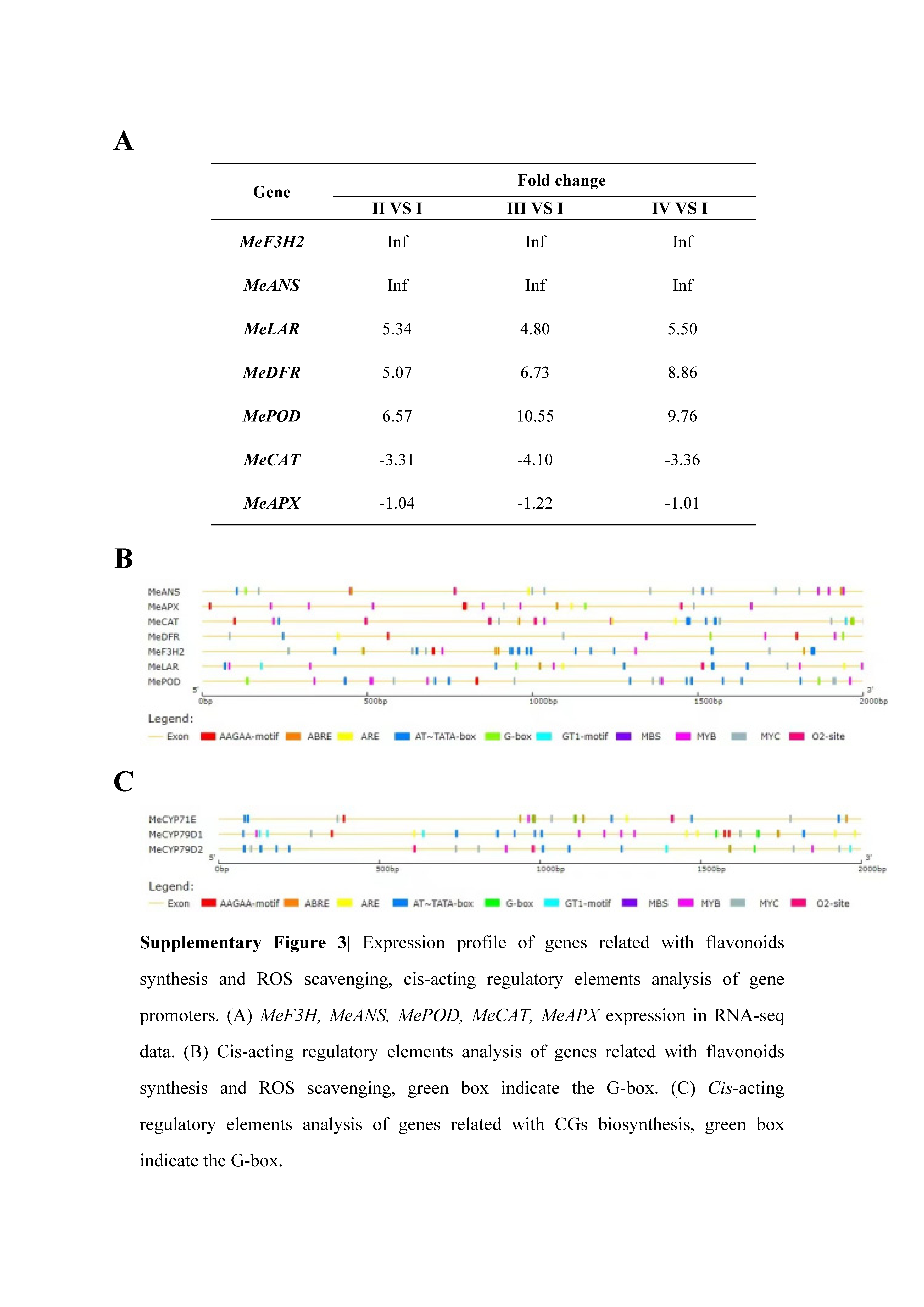

Supplement: Supplementary file 10 [file Image_3.TIFF]

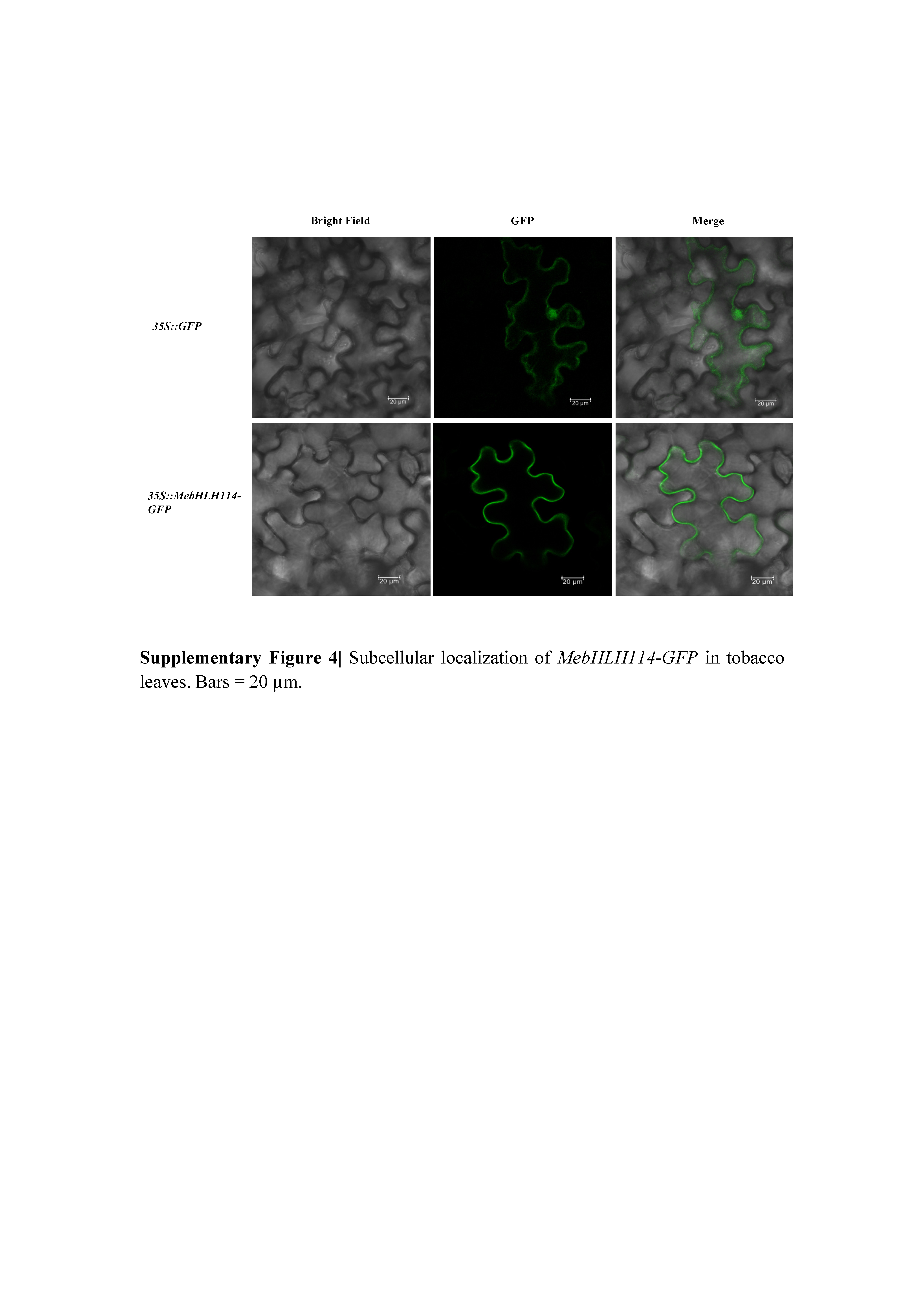

Supplement: Supplementary file 11 [file Image_4.TIFF]
